# Cytological and Pathological Correlation of FNAC in Assessing Breast Lumps and Axillary Lymph Node Swellings in a Public Sector Hospital in India

**DOI:** 10.1155/2013/695024

**Published:** 2013-12-17

**Authors:** Vasu Reddy Challa, Basavanna Goud Yale Guru, Poornima Rangappa, Vijayalakshmi Deshmane, devi. M. Gayathri

**Affiliations:** ^1^Department of Surgical Oncology, Kidwai Memorial Institute of Oncology, Bangalore, Karnataka 560029, India; ^2^Department of Pathology, Kidwai Memorial Institute of Oncology, Bangalore, Karnataka 560029, India

## Abstract

*Background*. Breast lumps have varied pathology, and there are different techniques to prove the diagnosis. The aim of the present study is to analyze the role of fine needle aspiration cytology (FNAC) of the breast lesions at our center. *Methods*. We had retrospectively analysed 854 patients who underwent FNAC for primary breast lumps and 190 patients who underwent FNAC for an axillary lymph node in the year 2010. *Results*. Of 854 patients, histological correlation was available in 723 patients. The analysis was done for 812 patients as medical records were not available for 42 patients. FNAC was false negative in seven cases; 2 cases of phyllodes were reported as fibroadenoma, and 5 cases of carcinoma were diagnosed as atypical hyperplasia. The sensitivity, specificity, and false negative value of FNAC in diagnosing breast lumps were 99% (715/723), 100%, and 1%, respectively. Of 190 patients for whom FNAC was performed for axilla, 170 had proven to have axillary lymph node metastases, and the rest had reactive hyperplasia or inflammatory cells. *Conclusions*. FNAC is rapid, accurate, outpatient based, and less complicated procedure and helps in diagnosis of breast cancer, benign diseases, and axillary involvement in experienced hands with less chance of false results.

## 1. Introduction

Breast cancer is one of the most common cancers in women in India and is a leading cause for mortality and morbidity. Breast tissue contains various tissue components, and there is change in composition of breast tissue with hormonal changes. So, it has varied pathology, and there are various modalities to prove the diagnosis. It is important for the clinician to examine properly and take proper decision during evaluation of a patient with palpable breast mass.

Triple assessment of breast mass had decreased the false negative rate to less than 1% [1]. But FNAC can be done as an outpatient procedure and helps in rapid diagnosis. The limitations of FNAC include difficulty to differentiate ductal carcinoma in situ (DCIS), atypical ductal hyperplasia from low grade DCIS, and fibroadenoma from phyllodes tumor [2, 3]. Core biopsy is an effective means to diagnose breast lumps, but it is expensive, time consuming, and associated with complications like haematoma and rarely pneumothorax [4, 5].

Many institutes in the United States, Canada, and the United Kingdom have given up FANC for diagnosing breast lesions, and they routinely perform core biopsy. But in many developing countries and in some European countries, FNAC is still routinely performed for diagnosing breast lesions [6]. FNAC is a rapid, less invasive, and less traumatic procedure but needs an experienced pathologist for better results.

The purpose of our study is to evaluate the efficacy of FNAC in diagnosing breast lumps in our setting, and it is one of the largest studies reported in India [6].

## 2. Materials and Methods

A retrospective analysis of medical records of the patients with detected breast mass (palpable and nonpalpable) and palpable axillary lymph node who were evaluated by FNAC at our centre in the year 2010 was valuated. Histopathological correlation was done wherever available. The procedure was performed by a pathologist using 21-22 Gauge needle with 2–4 aspirations and it was repeated if the sample was inadequate. For impalpable, and deeply located small lumps, ultrasound guided FNAC was performed. FNAC was done only for palpable lymph nodes. Sensitivity, specificity, and false negative and false positive values of FNAC for both breast lump and axilla were calculated. Statistical software SPSS 15.0 and MedCalc 9.0.1 were used to perform descriptive statistics.

## 3. Results

In a period of one year, 854 patients underwent FNAC of the primary breast lumps. As records of 42 patients were not available, they were excluded from the study. Hence, for 812 cases, histological correlation was available in 727 patients. The mean age of patients who underwent FNAC was 37.2 years (range 16–82 years). Ten male patients had undergone FNAC, two had gynaecomastia, and 8 had malignancy. Malignancy was the most common pathology of the patients who underwent fine needle aspiration cytology constituting 57% of all cases. The cytology reports were classified as benign, atypical, suspicious, malignancy, and unsatisfactory (Table 1).

**Figure 1:**
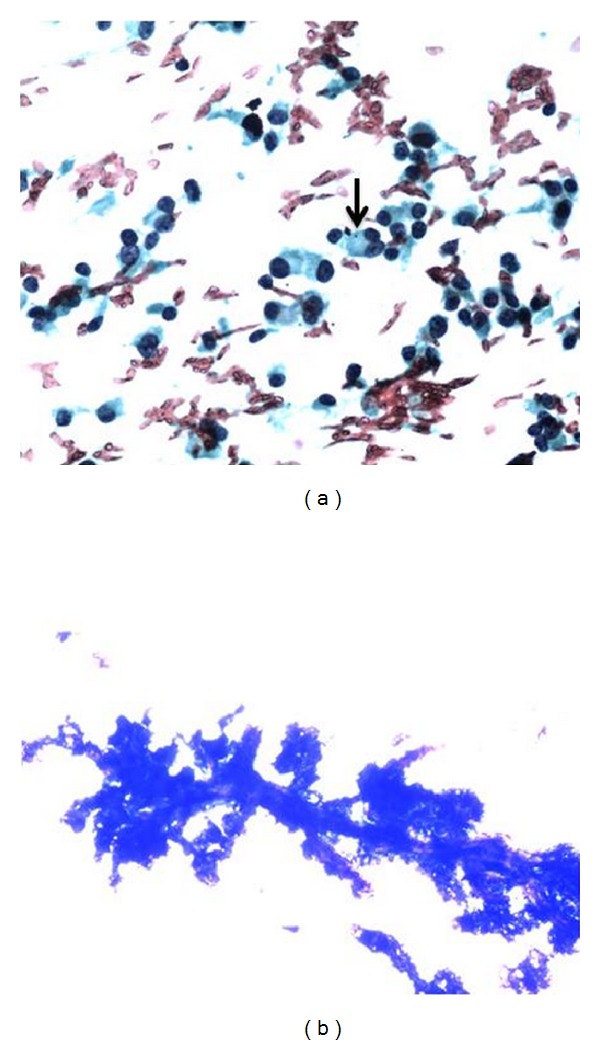
(a) FNAC breast lump showing PAP staining (20x) showing dispersed mildly pleomorphic plasmacytoid cells with azurophilic granules (arrow). Nucleus is irregular with some showing prominent nucleoli suggestive of neuroendocrine carcinoma. (b) FNAC breast lump with MGG staining (4x) showing papillary fragment with branching fibrovascular core suggestive of papillary neoplasm.

**Figure 2:**
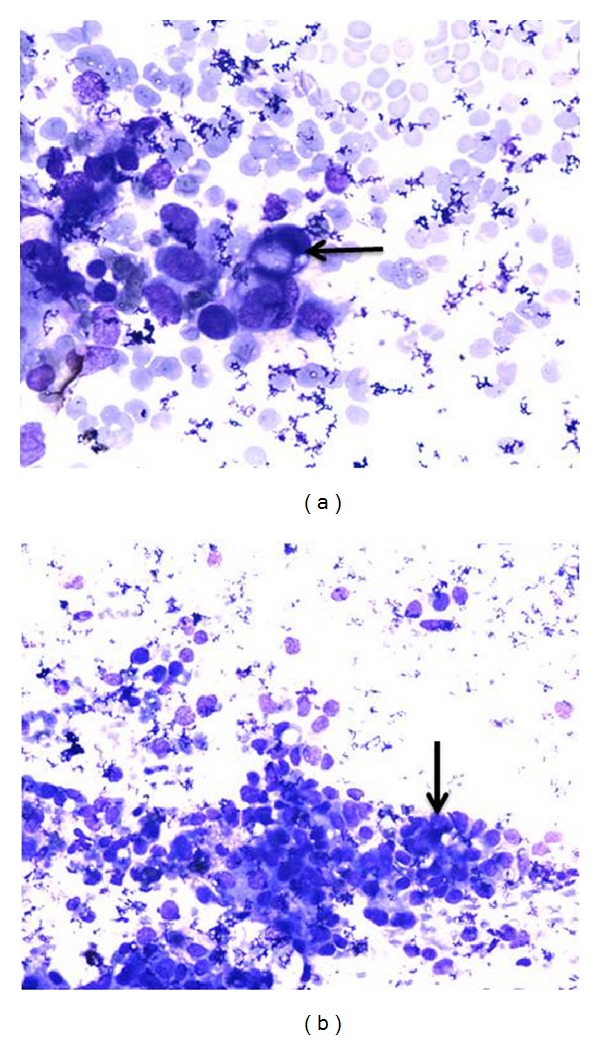
(a) FNAC breast lump with MGG staining (20x) showing signet ring cell (arrow). (b) FNAC breast lump with MGG staining (20x) showing acinar formation (arrow). It was diagnosed as metastatic lesion and proved to be gastric cancer metastasizing to breast.

Of the benign lesions, fibrocystic disease was the most common cytological diagnosis in 21% of patients (171 cases). Fibroadenoma was diagnosed in 98 patients with histology correlating with cytology in 96 cases. Two cases of phyllodes tumor were incorrectly reported as fibroadenoma on cytology. Of 27 cases who were diagnosed to have atypical lesions, (12 cases were papillary neoplasm, and 15 cases were atypical ductal hyperplasia) the final histopathology showed 12 cases of papillary carcinoma, 10 cases of papilloma, 10 had atypical ductal hyperplasia, 3 cases had ductal carcinoma in situ, and 2 cases had DCIS with a foci of invasive carcinoma. Cytology was useful in diagnosing various malignancies like neuroendocrine cancer, papillary neoplasms, and metastatic neoplasm from stomach (Figures 1 and 2).

Ten patients underwent ultrasound guided FNAC for deeply seated small lesions, of which 2 had invasive cancer, one was a case of papillary lesion later confirmed as papilloma, 2 were inflammatory lesions, and 5 patients had fibrocystic disease.

**Figure 4:**
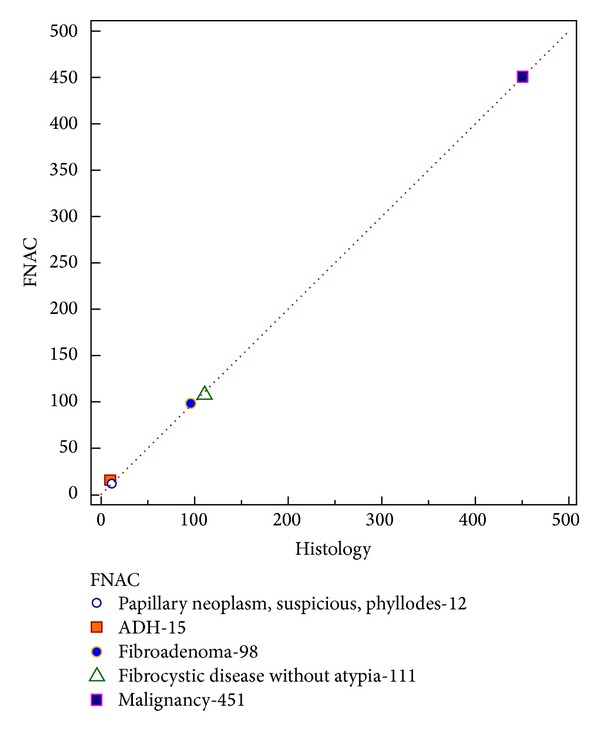
A concordance correlation coefficient scatter diagram of FNAC and histology showing the distribution of cases.

Of 8 patients with unsatisfactory reports, 4 patients were less than 30 years of age and were kept under followup, and clinicoradiologically, the lump appeared benign. The other 4 patients, though the FNAC was unsatisfactory due to discordance with clinicoradiological findings, were considered for excision, and one of the four patients had invasive ductal carcinoma. Seven cases were incorrectly diagnosed by FNAC (2 cases of phyllodes reported as fibroadenoma and 5 cases of carcinoma diagnosed as atypical hyperplasia). The sensitivity, specificity, and false negative value of FNAC in diagnosing breast lumps were 99% (715/723), 100%, and 1%, respectively (excluding patients with unsatisfactory samples) (Table 2). A concordance correlation coefficient was 0.99 (95% CI—0.9996 to 1.0000), and the Pearson coefficient was 0.99 which showed very good agreement between FNAC and the final histology (Figure 4).

### 3.1. FNAC—Fine Needle Aspiration Cytology

FNAC for a palpable axillary node was done in 190 patients of whom 170 had proven axillary lymph node metastases; the rest had reactive hyperplasia or inflammatory cells in the final histology. The sensitivity, specificity, and false positive and false negative results to rule out axillary lymph node metastases in a palpable lymph node were 90% (153/170), 100%, 0%, and 10% (17/170) (Tables 3 and 4).

## 4. Discussion

FNAC was first described by Martin and Ellis in 1930 for sampling cervical lymph nodes [7]. FNAC is a simple rapid technique performed as an outpatient procedure with less chance of complications unlike core biopsy which has risk of bleeding and occasionally rare complications like pneumothorax. It does not require any anesthesia or hospitalization and is cost effective. Experience and expertise in sampling and interpretation of specimen decide the effectiveness of FNAC. The other limitation of FNAC is it cannot differentiate few lesions like fibroadenoma from phyllodes, phyllodes from metaplastic carcinoma breast, papilloma from papillary carcinoma, and atypical ductal hyperplasia from ductal carcinoma in situ. Though a triple assessment is advocated for all palpable breast lumps, it is not feasible in developing countries like India, where affordability and availability are an issue at all centers and clinical decision takes a major role.

The sensitivity and specificity varied in different studies based on whether the unsatisfactory samples were considered positive or negative [6]. If the unsatisfactory samples were excluded from the study, the sensitivities and specificities varied from 58.3% to 100% and 55 to 100% [6]. Because an unsatisfactory sample needs further evaluation, few studies had considered them as a positive result and analysed there results. A meta-analysis of FNAC of breast lesions showed a sensitivity and specificity of 0.920 (95% CI, 0.906 to 0.933) and 0.768 (95% CI, 0.751 to 0.784) respectively (if the unsatisfactory samples were considered positive) [6]. If unsatisfactory samples were excluded from the study, the sensitivity was 0.927 (95% confidence interval [CI], 0.921 to 0.933); specificity 0.948 (95% CI, 0.943 to 0.952); positive likelihood ratio 25.72 (95% CI, 17.35 to 28.13); negative likelihood ratio 0.08 (95% CI, 0.06 to 0.11); diagnostic odds ratio 429.73 (95% CI, 241.75 to 763.87) (Table 5). Also, in these meta-analyses, when the pooled unsatisfactory samples were further analyzed, there was upgrading to cancer in 27.5% of cases. In our study, there was one of 8 cases (12.5%) who had been upgraded to cancer in unsatisfactory samples. We had 7 false negatives, 2 cases reported as fibroadenoma preoperatively turned out to be phyllodes, and 5 cases of carcinoma were diagnosed as atypical hyperplasia preoperatively.

The most common reason for a false negative result is failure to localize the lesion exactly. This can be overcome by performing the test under image guidance [8]. Also, tumors with extensive fibrosis like scirrhous carcinoma can give a false negative result because of low cellularity which can be avoided by performing FNAC in the periphery of the tumor with 27-gauge needle in this type of malignancies [9]. The false positive results occur because of some benign diseases with hypercellularity like cellular fibroadenoma, proliferative fibrocystic disease, phyllodes tumor, lactational changes, sclerosing adenosis, and so forth, and in lesions with atypia like postradiation, fat necrosis, and radial scar [9]. False positives can be reduced by considering for biopsy in patients fulfilling DeMay's criteria in cases with (a) poor cellular aspirate, (b) absent single intact cells, (c) presence of bare bipolar nuclei, and (d) absent/less amount of atypia [10].

For no palpable lymph nodes, ultrasonography with FNAC as a staging tool avoided sentinel lymph node biopsy in 68% of patients (upfront underwent axillary lymph node dissection) with positive axillary lymph nodes [11]. Roles of FNAC in palpable lymph nodes to rule out metastases were evaluated in a study which showed diagnostic accuracy for metastatic lymph nodes as 97.9%, sensitivity of 97.9%, and specificity of 100% [12]. Our study is in well correlation with literature with sensitivity of 90% in diagnosing axillary lymph node involvement. The cost effectiveness of FNAC of breast was studied in a study by Silverman et al. They demonstrated less costs with FNAC of breast and helped to triage the patients to either outpatient or inpatient setting [13]. This is important in poor resource setting like our hospital where most of the patients present in advanced stage and cannot afford expensive investigations. We would like to bring in light that FNAC is still an acceptable mode of diagnosis in experienced hands and where there is limitation of resources.

## 5. Conclusion

FNAC is a safe, rapid, and effective test for diagnosing breast lumps in experienced hands. Unsatisfactory samples should not be neglected and should be considered for excisional biopsy in presence of clinical suspicion.

## Figures and Tables

**Figure 3:**
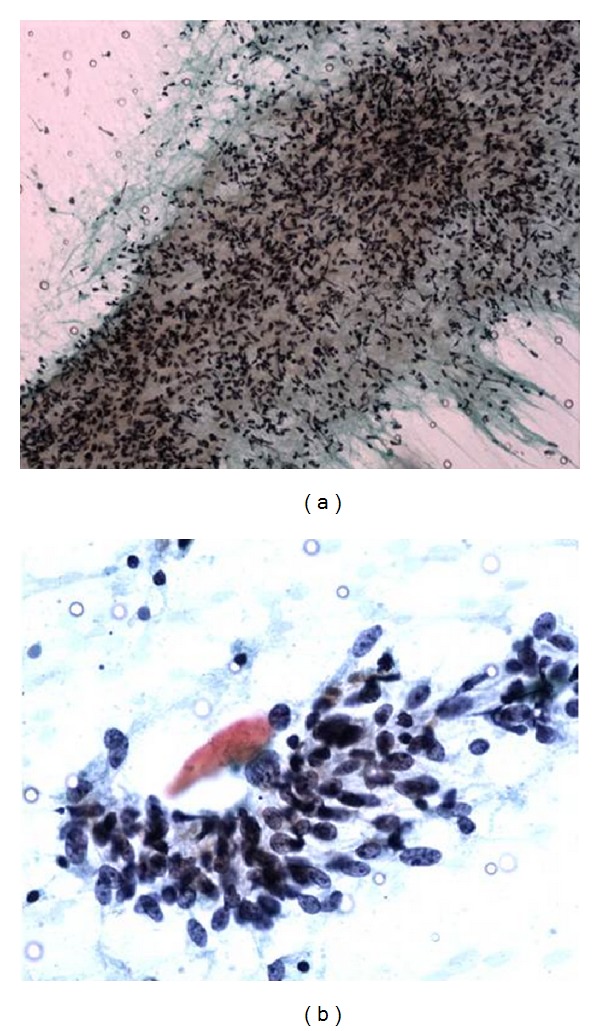
(a) PAP staining (20x) showing hypercellular spindle cell stromal component. Moderate nuclear pleomorphism is evident. (b) PAP staining (40x) showing loose cohesive cluster of epithelial cells with moderate nuclear pleomorphism suggestive of phyllodes tumor.

**Table 1:** Cytology impression of the patients who underwent FNAC of breast lumps.

Report	Total number of patients (812)
Benign	314 (38.6%)
Benign lesions with no risk of cancer	83 (10.2%)
Benign proliferative disease without atypia	231 (28.4%)
Atypical	27 (3.3%)
Suspicious	12 (1.4%)
Malignancy	451 (55.5%)
Unsatisfactory	8 (0.9%)

**Table 2:** Comparison of cytology and histopathology of breast lesions.

Type of lesion	No. of cases (812)	Patients with histological correlation (727)
(A) Benign lesions with no risk of cancer	**83 (10.2%)**	**12**
(1) Inflammatory breast lesions	**27 (3.3%)**	
(a) Breast abscess	5 (0.6%)	—
(b) Granulomatous mastitis	12 (1.4%)	2
(c) Fat necrosis	5 (0.6%)	—
(d) Periductal mastitis/duct ectasia	5 (0.6%)	—
(2) Nonproliferative breast disorder	**40 (4.9%)**	
(a) Simple cyst	10 (1.2%)	—
(b) Fibrocystic change	30 (3.6%)	10
(3) Miscellaneous breast lesions	**16 (1.9%)**	
(a) Galactocele	2	—
(b) Lipoma	3	—
(c) Gynaecomastia	2	—
(d) Axillary breast tissue	3	—
(e) Suture granuloma	2	—
(f) Hamartoma	2	—
(g) Diabetic mastopathy	1	—
(h) Radiotherapy induced mastitis	1	—

(B) Benign lesions with mild to moderate risk of cancer	**258 (31.7%)**	**248**
(1) Proliferative breast disease without atypia	**231 (28.4%)**	**221**
(a) Fibrocystic disease without atypia (Moderate epithelial hyperplasia and adenosis)	121 (14.9%)	111
(b) Fibroadenoma	98 (12%)	98 (2 cases turned as phyllodes)
(c) Phyllodes tumor Figure 3	12 (1.4%)	12
(2) Proliferative breast disorder with Atypia	**27 (3.3%)**	**27**
(a) Atypical ductal hyperplasia	15 (1.8%)	15 (5 cases turned as carcinoma)
(b) Papillary neoplasm	12 (1.4%)	12 (10 cases papilloma, 2 cases papillary carcinoma)

(C) Suspicious and malignant lesions	**463 (57%)**	463
(1) Suspicious	12 (1.4%)	12
(2) Carcinoma	449 (55.2%)	449
(3) Malignant myoepithelioma	1	1
(4) Metastatic adenocarcinoma	1	1

(D) Unsatisfactory	8	4 (1 case invasive ductal carcinoma)

**Table 3:** FNAC and histology correlation of axillary lymph node status.

	Axillary lymph node metastases present	Axillary lymph node metastases absent
FNAC positive	153	0
FNAC negative	17	20

**Table 4:** Diagnostic performance of FNAC axillary lymph node status (*N* = 190).

(i) Sensitivity	90% (153/170)
(ii) Specificity	100% (20/20)
(iii) False positive	0%
(iv) False negative	10% (17/170)

**Table 5:** Summary of studies showing the results of FNAC (after excluding unsatisfactory samples).

Author (year)	Number of patients	Sensitivity	Specificity	False positive	False negative
Tao et al. (2004) [14]	2701	97.9%	99.8%	0.2%	2.1%
Yu et al. (2006) [15]	2128	98%	75.5%	24.5%	2%
Wang (1981) [16]	1024	88.1%	91.7%	8.3%	11.9%
Farshid et al. (2008) [17]	1093	97.8%	88%	12%	2.2%
Vasu et al. (present study)	723	99%	100%	0%	1%

## List of Abbreviations

FNAC: Fine needle aspiration cytology
DCIS: Ductal carcinoma in situ
PAP: Papanicolaou
MGG: May-Grünwald Giemsa stain.
